# A Flexile and High Precision Calibration Method for Binocular Structured Light Scanning System

**DOI:** 10.1155/2014/753932

**Published:** 2014-08-18

**Authors:** Jianying Yuan, Qiong Wang, Bailin Li

**Affiliations:** ^1^School of Mechanical Engineering, Southwest Jiaotong University, Chengdu, Sichuan 610031, China; ^2^School of Automatic Control and Mechanical Engineering, Kunming University, Kunming, Yunmun 650214, China

## Abstract

3D (three-dimensional) structured light scanning system is widely used in the field of reverse engineering, quality inspection, and so forth. Camera calibration is the key for scanning precision. Currently, 2D (two-dimensional) or 3D fine processed calibration reference object is usually applied for high calibration precision, which is difficult to operate and the cost is high. In this paper, a novel calibration method is proposed with a scale bar and some artificial coded targets placed randomly in the measuring volume. The principle of the proposed method is based on hierarchical self-calibration and bundle adjustment. We get initial intrinsic parameters from images. Initial extrinsic parameters in projective space are estimated with the method of factorization and then upgraded to Euclidean space with orthogonality of rotation matrix and rank 3 of the absolute quadric as constraint. Last, all camera parameters are refined through bundle adjustment. Real experiments show that the proposed method is robust, and has the same precision level as the result using delicate artificial reference object, but the hardware cost is very low compared with the current calibration method used in 3D structured light scanning system.

## 1. Introduction

**Figure 1:**
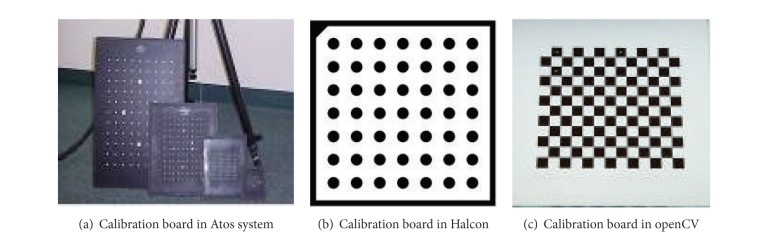
Calibration of 2D board patterns commonly used.

Binocular structured light scanning system (BSLSS) is widely used in the fields of reverse engineering [1], inspection [2], medical analysis [3], and human body motion analysis [4] due to its advantages of noncontact, high speed, and high precision [5]. The system principle is based on binocular stereo vision, and usually it is composed of two digital cameras and a commercial DLP projector [6]. Before scanning, camera parameters should be calibrated, which include intrinsic parameters (effective focal length, principle point, and lens distortion coefficient) and extrinsic parameters (orientation of two camera coordinate frames relative to a certain world coordinate frame). The process of calibration is not only the first step of scanning but also has great influences on scanning precision, which is critical to the overall system and has been studied extensively in computer vision, and even recently new techniques have been proposed in the papers [7–9]. In BSLSS (binocular structured light scanning system), in order to get high precision, 3D or 2D elaborate reference object is usually required in the papers [10–12]. The 3D calibration object usually consists of two or three orthogonal planes with each other, and 2D calibration reference object is usually a plane with some patterns on it, as shown in Figure 1; the patterns are circle and chessboard. The adjacent circle center distance or chessboard corner should be known in advance. In the calibration, the planar pattern should be imaged at a few different orientations, as in Aots system; 18 orientations are necessary. Although the calibration algorithm based on 3D or 2D object can achieve high precision which can meet the need of industrial measuring, there are two drawbacks. One is that high precision calibration reference objects need the fine processing, so it is usually expensive. The other is that it is not suitable for large field of view. In this paper, we put forward a novel camera calibration method, which is based on hierarchical self-calibration and bundle adjustment. Hierarchical self-calibration means that we estimate extrinsic parameters though different spaces, more specifically, we first get the initial extrinsic parameters of the cameras in the projective space using factorization method and then upgrade to Euclidean space with orthogonality of rotation matrix and rank 3 of the absolute quadric as constraint. Bundle adjustment method is an optimize technique and widely used in photogrammetry. In this paper, we use this technique to refine all the parameters to guarantee that high precision calibration can be achieved. The novel method is free of fine processing and complex mechanical control setup, only a few of small size patterns printed on PVC membrane and a stand scale bar are needed, which are very easy to process and the cost is much lower than that of current plane board. Moreover, the algorithm proposed is suitable for calibration of large field of view. Real experiments have given out the reliability and validity of this method.

## 2. Algorithm in This Paper 

### 2.1. Hardware Used

**Figure 2:**
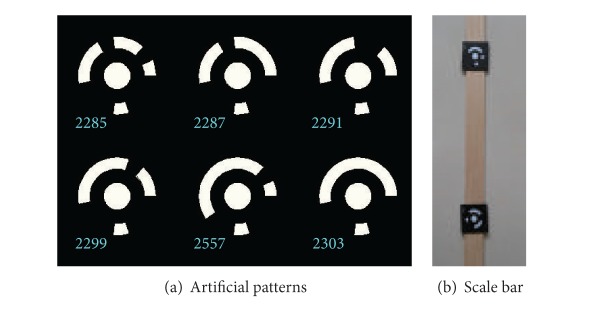
Patterns and scale bar used in this paper.

Like the traditional method proposed by Zhang and Tsai, the method in this paper also needs to build the relationships between space points and their projection in image. So space points and their projections should be defined firstly. In this paper, some patterns with a unique ID number are designed for space object points. The patterns are a white circle with circular sections around it, just as shown in Figure 2(a). The white circle center is used as a space object point, and their projection on image can be acquired by circle center detection. The circular sections give ID information of the circle. The scale bar is a bar with two coded circles whose distance is detected by the third measuring system.

### 2.2. Binocular Vision Model and Parameters to be Calibrated

**Figure 3:**
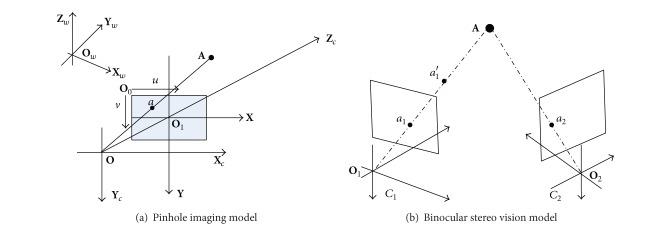
Pinhole imaging and binocular stereo vision model.

This section describes the binocular vision model and defines the calibration parameters.  Figure 3(a) illustrates the pinhole imaging model of one camera. A 3D space point in denoted by **A** = [*x* *y* *z* 1]^*T*^, and its corresponding image point is denoted by *a*(*u*, *v*). **O**_*w*_ **X**_*w*_ **Y**_*w*_ **Z**_*w*_ is the world coordinate system, and **O**_*c*_ **X**_*c*_ **Y**_*c*_ **Z**_*c*_ is the camera coordinate system. **O**_0_ *uv* is the pixel coordinate system. **O** is the optical center of camera. The mathematical model between **A** and *a* can be formulated as

(1) $$\begin{array}{c} \lambda \left[\begin{array}{c} u\\ v\\ 1 \end{array}\right]=PA, \end{array}$$

where (*u*, *v*) is the coordinate of *a* in the image plan, **P** = **K**(**R** **T**), *λ* represents the projection depth, and **K** is camera interior parameter matrix defined as

(2) $$\begin{array}{c} K=\left[\begin{array}{ccc} f_{u} & s & u_{0}\\ 0 & f_{v} & v_{0}\\ 0 & 0 & 1 \end{array}\right], \end{array}$$

where *f*_*u*_ and *f*_*v*_ are the scale factors in image *u* and *v* axes; *s* is the parameters describing the skewness of the two image axes and is often the zero; (*u*_0_, *v*_0_) is the coordinates of principle points which would be ideally in the centre of the image; **R** is the rigid body transformation from the world coordinate system to the camera 3-D coordinate system (which is also called rotation matrix). **T** is the translation vector. **R** and **T** are extrinsic parameters.

The binocular vision geometric model is illustrated in Figure 3(b). *C*_1_ and *C*_2_ are the two optical centers, *a*_1_, *a*_2_ are the corresponding image points of space point **A**. The left and right camera imaging equations are described as follows:

(3) $$\begin{array}{c} \lambda_{l}\left[\begin{array}{c} u_{l}\\ v_{l}\\ 1 \end{array}\right]=K_{l}\left(R_{l}\;|\;T_{l}\right)A,\\ \lambda_{r}\left[\begin{array}{c} u_{r}\\ v_{r}\\ 1 \end{array}\right]=K_{r}\left(R_{r}\;|\;T_{r}\right)A. \end{array}$$

In the above two sets of equations, four equations are independent, and the 3D coordinate of a space object point can be solved through using a least squares estimator. We can assume that the world coordinate and left camera coordinate is the same, which means **R**_*l*_ = **I**, **T**_*l*_ = 0, where **I** is an identity matrix and 0 is a zero vector. Therefore, in order to solve the 3D coordinate of a space object point, parameters to be calibrated include intrinsic parameters of the left and right cameras, which are defined as **K**_*l*_, **K**_*r*_, and external parameters of the right camera, can be defined as **R**_*r*_, **T**_*r*_.

### 2.3. The Algorithm Flow

**Figure 4:**
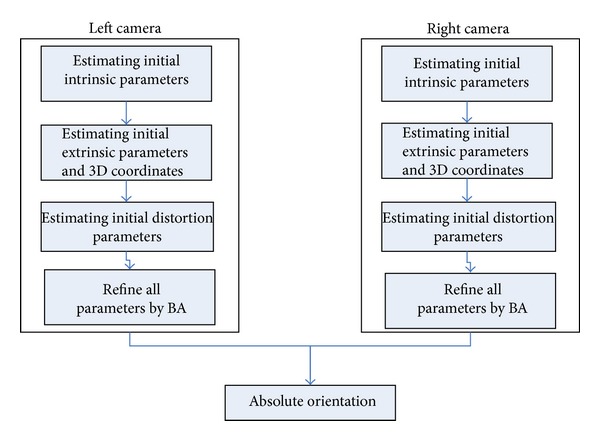
Algorithm flow.

Our aim is to get the intrinsic parameters and relative position relationship of two cameras used in BSLSS; the proposed algorithm flow is illustrated in Figure 4.

Left and right cameras are calibrated under its own world coordinate **O**_*w*_ **X**_*w*_ **Y**_*w*_ **Z**_*w*_ separately first then absolute oriented to a global world coordinate, and the global world coordinate is the left camera coordinate. The process of calibration of a signal camera can be divided into four steps. First step is to make estimating initial intrinsic parameters from image and the second step is to estimate initial extrinsic parameters and 3D objects based on hierarchical self-calibration. Third step is estimating initial distortion parameters through corresponding image points and space 3D points. At last, all the parameters are refined through bundle adjustment.

### 2.4. Calibration of One Camera in Its Own World Coordinate

#### 2.4.1. Estimating Initial Intrinsic Parameters of One Camera

The intrinsic parameters encompass focal length, image format, and principle point, which are the camera's intrinsic properties and they are not to be changed. So, we can compute the intrinsic parameters in advance. Here, we provided two methods to get the intrinsic parameters. One is using a panel to compute intrinsic parameters. When imaging a panel, there is a homography matrix linking the image panel and the space panel; intrinsic parameters can be estimated from the homography matrix. The principle of this method can be found in the paper [11]. The other method is very simple, and it can be assumed that there is little error in the camera assembly process. Since the intrinsic parameters contain the intrinsic properties of the camera, those parameters can be obtained from camera operation manual which the camera manufacturers will provide. More specifically, those parameters are set as following: *s* = 0, *u*_0_ = width/2, *v*_0_ = hight/2, *f*_*u*_ = *f*_*v*_ = (*f*/*dx* + *f*/*dy*)/2, where *f* is the focal length, *dx* and *dy* are the center to center distance between adjacent sensor elements in **X** direction and **Y** direction, width is the width of image in pixel, and height is the height of image in pixel.

#### 2.4.2. Estimating Initial Extrinsic Parameters of One Camera

The process of obtaining initial value of camera extrinsic parameters can be divided into three steps. The first step is to get camera motion and scene sharp matrix in projective space with the method of factorization, and the second step is to get camera motion and scene sharp matrix in Euclidean space. The third step is to get the initial value of camera external parameters by decomposing the camera motion matrix.

In the first step, supposing there are *p* 3D space objects visible in *F* images, the rescaled measurement matrix **W**_3*F*×*p*_ can be got, and **W**_3*F*×*p*_ has rank at most 4. **W**_3*F*×*p*_ can be divided into **Q** and **X**, described as (4), where *λ*_*ij*_ is the projective depth, **X**_*j*_ is the unknown homogeneous coordinate vectors of the 3D points called sharp matrix, **P**_*i*_ is the unknown 3 × 4 image projection matrix also called motion matrix in some other articles, and *m*_*ij*_ is the measured homogeneous coordinate vectors of the image points, where *j* = 1 ⋯ *p* labels points and *i* = 1 ⋯ *F* labels images. Each object is defined only up to an arbitrary nonzero rescaling. Consider

(4) $$\begin{array}{c} \underset{W}{\underset{︸}{\left[\begin{array}{ccc} \lambda_{11}m_{11} & \cdots & \lambda_{1p}m_{1p}\\ & \ddots & \\ \lambda_{F1}m_{F1} & \cdots & \lambda_{Fp}m_{Fp} \end{array}\right]}}=\underset{Q}{\underset{︸}{\left[\begin{array}{c} P_{1}\\ P_{F} \end{array}\right]}}\underset{X}{\underset{︸}{\left[\begin{array}{c} \begin{array}{ccc} X_{1} & \cdots & X_{p} \end{array} \end{array}\right]}}. \end{array}$$

Through method of SVD decomposing, we can get **Q** and **X**, and the premise is that we know the projective depth of each image point. There are two methods to estimate projective depth, one is based on fundamental matrices and epipoles, and the other is based on minimizing the rank of **W**_3*F*×*p*_ to 4. The detail of estimating projective depth can be found in the papers [13, 14]. In this paper, we estimate projective depth by the algorithm proposed in the paper [13]. The principle of this method is based on epipolar geometry, and the solving of *λ*_*ij*_ can be got through the following:

(5) $$\begin{array}{c} \lambda_{ip}=\frac{\left(e_{ij}\wedge q_{ip}\right)\cdot \left(F_{ij}q_{jp}\right)}{{||e_{ij}\wedge q_{ip}||}^{2}}\lambda_{jp}, \end{array}$$

where *F*_*ij*_ is the fundamental matrix between the *i*th and *j*th images, which satisfy the constraint of *q*_*j*_^*T*^ *F*_*ij*_ *q*_*i*_ = 0; *e*_*ij*_ is the pole point on the *i*th image, and *q*_*ip*_ and *q*_*jp*_ are the corresponding image point coordinates on the *i*th and *j*th image separately. When estimating *λ*_*ij*_, we assume that the projective depths of every reference image point is 1, *λ*_1*p*_ = 1 first, and then substitute it to (5) to get all the other projectiave depth on other images.

The second step is that, in European space, the projective matrix of one image can be expressed as **P**_*Ei*_ = **K**_*i*_ (**R**_*i*_∣**T**_*i*_), and we can get following through considering m images:

(6) P1H=α1PE1= α1K1(R1 ∣ T1)⋮PmH=αmPEm= αmKm(Rm ∣ Tm),

where **P**_*i*_ is the projection matrix in projective space and *α*_*i*_ is an arbitrary nonzero number. **H** is a transformation matrix. The main task in the second step is to solve **H**, thus **P**_*Ei*_ can be got. Because the rotation matrix is an orthogonal matrix, which means **R**_*i*_ **R**_*i*_^*T*^ = **I**, the following can be deduced through using this property:

(7) P1H0H0TP1T= α12K1K1T⋮PiH0H0TPiT= αi2KiKiT,

where **H**_0_ is the first three columns of **H** and **K**_1_ = ⋯ = **K**_*i*_ = **K**. Set Tran = **H**_0_ **H**_0_^*T*^, and Tran is a 4 × 4 symmetric matrix with 10 unknowns. One unknown factor will be added when one image is added, so in order to solve Tran, at least three images are needed [15]. Meanwhile, Tran represents a dual absolute quadric surface, with a rank of three and det⁡(Tran) = 0. This is a constraint to solve Tran. In order to guarantee the rank of Tran is three, SVD decomposition for Tran is carried out and the fourth singular value is seted zero. As shown in (8), **H**_0_ can be got, where **A**_0_ is the first three columns of **A**. Consider

(8) Tran=Adiag⁡(σ1,σ2,σ3,0)AT=Adiag⁡(σ1,σ2,σ3,0)×diag⁡(σ1,σ2,σ3,0)TAT⇓H0=A0diag⁡(σ1,σ2,σ3).

The method to solve the fourth column of **H** is described as follows. Assuming *x*_0_ is one of the points in **Q** and it is also the origin point of European space coordinate system, then **H** ^−1^ *x*_0_ = (0 0 0 1)^*T*^ can be obtained, yielding **H**_4_ = *x*_0_. At this point, all the elements in **H** are solved. Motion and shape matrixes in European space are represented as follows:

(9) $$\begin{array}{c} P_{e}=PH,\\ Q_{e}=H^{-1}Q. \end{array}$$

In the above deducing process, we do not make any assumptions on the intrinsic parameters, and the rank of dual absolute quadric surface is three and is used as constraint factor which guarantees the robustness of the solving process.

The third step is that if **P**_*ei*_ = **P**_*i*_ **H**, **X**_*ei*_ = **H** ^−1^ **X**_*i*_, **R**_*i*_ **T**_*i*_ can be solved by decomposing **P**_*ei*_ through method of **Q** **R** decomposition.

#### 2.4.3. Estimating Initial Distortion Coefficients of One Camera

The image captured by the digital camera is not satisfied with the pinhole camera model, so we should consider lens distortion of the camera. In this section, we only consider the first two terms of radial distortion. The coefficients of the radial distortion can be solved through the following:

(10) u′=u+(u−u0)[k1(x2+y2)+k2(x2+y2)2],v′=v+(v−v0)[k1(x2+y2)+k2(x2+y2)2],

  (*u*, *v*) represents the ideal pixel image coordinates, which can be obtained through (1). (*u*′, *v*′) represents the corresponding real observed image coordinates, which can be obtained through image feature detection algorithm. (*x*, *y*) denotes the ideal normalized image coordinates; the solving for it can be found in the paper [11]. (*u*_0_, *v*_0_) denotes the principle points. (*k*_1_, *k*_2_) denotes the coefficients of the radial distortion.

#### 2.4.4. Refine All Parameters of One Camera Using Bundle Adjustment

In the above Sections 2.4.1 to 2.4.3, the solving of initial intrinsic, extrinsic parameters and distortion coefficients is completed. Usually, the precision is not very high, so in order to achieve high calibration precision, bundle adjustment method is adopted to refine all the parameters. Bundle adjustment method is an optimization technique originally conceived in the field of photogrammetry and has increasingly been used by vision researches during the last decade. The optimized objective function is as follows:

(11) $$\begin{array}{c} \min\overset{n}{\underset{i=1\;}{\sum }}\overset{m}{\underset{j=1}{\sum }}d{\left(x_{ij}\left({Angle}_{ei},t_{ei},K,Q_{ej}\right),q_{ij}\right)}^{2}, \end{array}$$

where *x*_*ij*_ is the reprojective point of the *j*th space point on the *i*th camera, **A** **n** **g** **l** **e**_*ei*_ is a vector that represents the Euler angle of the *i*th camera, **t**_*ei*_ is the translation vector, **Q**_*ej*_ is the *j*th control points, and *q*_*ij*_ is the *j*th image point on the *i*th camera. More details about BA can be found in the paper [16].

### 2.5. Unifying Extrinsic Parameters of Left and Right Camera to Global Coordinate

Only the parameters of single camera are solved in the analysis process mentioned above. The global coordinate of the two cameras is not the same. In this section, we will give the algorithm of unifying the two different coordinates of the two cameras into one global coordinate.

Assuming **X**_*l*_ and **X**_*r*_ are 3D points in left and right camera world coordinate, and the corresponding projective matrixes are **P**_*l*_ and **P**_*r*_ separately. According to the principle of pinhole imaging, the image point and the object point will be satisfied with the following equation, where $\begin{array}{cc} \left(u_{l},v_{l}\right) & \left(u_{r},v_{r}\right) \end{array}$ are image points in left and right images:

(12) [ulvl1]T=PlXl,[urvr1]T=PrXr=Pr′Xl.

The relationships between **X**_*l*_ and **X**_*r*_ are illustrated as in (6), where **G**_4×4_ is a rigid transformation matrix. Consider the following:

(13) $$\begin{array}{c} X_{r}=G_{4\times 4}X_{l}. \end{array}$$

From (12) and (13), we can calculate **P**_*r*_′ = **P**_*r*_ **G**_4×4_. And then a unified coordinate is obtained and thus the calibration is completed.

## 3. Experimental Results

**Figure 5:**
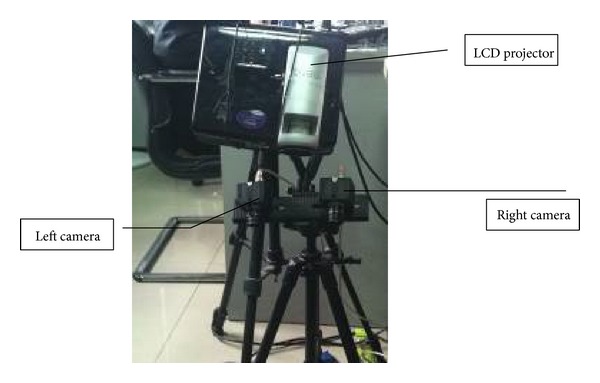
Hardware of binocular system.

**Figure 6:**
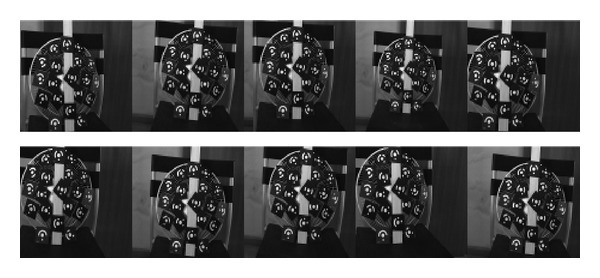
Artificial targets captured by the left and right cameras.

The experiments for testing our proposed method are given out in this section. The binocular structured light system, which is shown in Figure 5, consists of two digital cameras with a total resolution of 1280 × 1024 pixels and an LCD projector with resolution of 1024 × 768. The distance between two cameras is about 30 mm, and the measuring volume depends on the focusing capability and the field of camera's view. In our system, a lens with 6 mm is chosen, and the measuring volume is about 400 mm ∗ 300 mm ∗ 300 mm. Artificial square targets with a side length of 5 mm and a scale bar are placed in the measurement field randomly, which are captured simultaneously by the two cameras. The distance between targets and two cameras is about 1000 mm. 20 images from different positions and angles are used to solve parameters, which are as shown in Figure 6. (Only 10 images are shown due to limited space).

**Figure 7:**
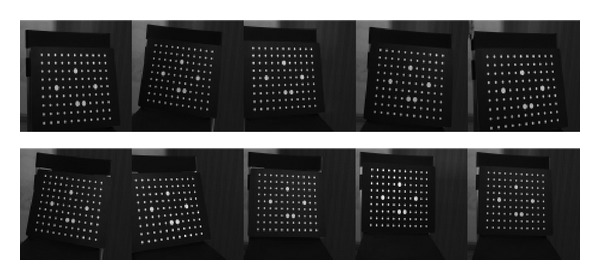
Panel board in Zhang's method captured by the left and right cameras.

Method proposed by Zhang [11] is the most widely used calibration method in current BSLSS. So, the performance of the method proposed is evaluated by comparing the results with respect to a classical panel calibration described by Zhang. The panel board used in Zhang's algorithm is as shown in Figure 7. The coordinates of circle center are measured through the third measurement, and the distance between two circle centers is known in advance.

The panel board used in Zhang's algorithm is also used as a standard object. When calibration is completed, we will use the calibration parameters to calculate the 3D distance between two circle centers. It is assumed that the *i*th true distance is *d*_*i*_, the same distance calculated by the method proposed and Zhang's method are *d*_*i*_′ and *d*_*i*_′′ separately. It can define mean distance error (MDE) *e*_1_ = (1/*n*)∑_*i*=1_^*n*^|*d*_*i*_ − *d*_*i*_′| and *e*_2_ = (1/*n*)∑_*i*=1_^*n*^|*d*_*i*_ − *d*_*i*_′′|, and through comparing *e*_1_ with *e*_2_, the performance of method can be analyzed.

In order to avoid random error, ten group experiments are carried out, and the results are as described in Table 1. In order to analyze the relationships between number of image and calibration precision, different number images are tested, which is also shown in Table 1. From Table 1, we can see that when the number of images is small (the number is 7), the calibration method in this paper is slightly lower than that of Zhang's method. When the number of images is increased to 14 or 20, the calibration precision is almost the same. One of the experimental results is shown in Table 2. From Table 2, we can see that the calibration results between Zhang's method and the proposed method are slightly different.

## 4. Conclusions

As the current camera calibration method in the BSLSS (binocular structured light scanning system) has high cost, In this paper, we put forward a novel calibration method which does not rely on complex calibration reference object, and the hardware are some small size artificial targets and a scale bar. Because the hardware used does not need the strict industrial processing, the cost is lower compared with traditional 2D or 3D elaborated object. Besides, the size of hardware used in this paper is very small which means they are more flexible than 2D or 3D calibration reference object. Real experimental results show that the calibration precision is the same as the traditional method when calibration images are enough.

## Figures and Tables

**Table 1:** Calibration error (mm).

Image number	Proposed method (*e*_1_)			Method proposed by Zhang (*e*_2_)		
	7	14	20	7	14	20
1	0.050	0.021	0.011	0.041	0.034	0.012
2	0.047	0.032	0.027	0.025	0.029	0.039
3	0.061	0.011	0.034	0.037	0.028	0.019
4	0.103	0.028	0.018	0.031	0.051	0.027
5	0.032	0.036	0.012	0.028	0.047	0.014
6	0.098	0.040	0.019	0.029	0.013	0.009
7	0.057	0.035	0.017	0.037	0.018	0.015
8	0.046	0.022	0.020	0.035	0.024	0.020
9	0.039	0.023	0.025	0.041	0.015	0.034
10	0.049	0.039	0.020	0.022	0.022	0.009

Average error	0.058	0.0287	0.0203	0.0326	0.0281	0.0198

**Table 2:** Calibration results of Zhang's method and the proposed method (image number = 10).

	Zhang's method			Method in this paper		
Intrinsic parameters						
Left camera	*f*_*u*_ = 2340.82; *u*_0_ = 590.32			*f*_*u*_ = 2340.817; *u*_0_ = 590.31		
	*f*_*v*_ = 2340.25; *v*_0_ = 548.90			*f*_*v*_ = 2340.25; *v*_0_ = 548.91		
Right camera	*f*_*u*_ = 2340.82; *u*_0_ = 597.25			*f*_*u*_ = 2323.27; *u*_0_ = 597.28		
	*f*_*v*_ = 2322.95; *v*_0_ = 544.33			*f*_*v*_ = 2322.97; *v*_0_ = 544.21		
Extrinsic parameters						
Rotation matrix	0.9493	−0.0047	−0.3144	0.9494	−0.0044	−0.3139
	0.0069	1.0000	0.0062	0.0061	1.0000	0.0042
	0.3144	−0.0081	0.9493	0.3139	−0.0059	0.9494
Translation vector	202.56768	0.272812	15.384653	201.8624	1.3684	15.5949
Distortion coefficients						
Left camera	*k*_1_ = −0.055243; *k*_2_ = 0.084703			*k*_1_ = −0.0430; *k*_2_ = 0.0304		
Right camera	*k*_1_ = −0.061157; *k*_2_ = 0.130932			*k*_1_ = −0.0432; *k*_2_ = −0.0005		
